# Parental Separation and Children’s Education—Changes Over Time?

**DOI:** 10.1007/s10680-024-09721-7

**Published:** 2025-01-20

**Authors:** Sanna Kailaheimo-Lönnqvist, Marika Jalovaara, Mikko Myrskylä

**Affiliations:** 1 University of Helsinki, Helsinki, Finland; 2 University of Turku, Turku, Finland; 3 Max Planck Institute for Demographic Research, Rostock, Germany; 4 Max Planck, University of Helsinki Center for Social Inequalities in Population Health (MaxHel), Rostock, Germany; 5 Max Planck, University of Helsinki Center for Social Inequalities in Population Health (MaxHel), Helsinki, Finland

**Keywords:** Parental separation, Parental divorce, Children’s education, Cohort differences

## Abstract

The association between parental separation and children’s education has been widely studied, but mainly at a single time point and for marital dissolution only. We examine whether the (generally negative) association has changed across cohorts for several educational outcomes and whether the association differs by parental union type (marriage, cohabitation) and socioeconomic family background (parental education).We use Finnish total population register data. We focus on child cohorts born between 1987 and 2003 (*N* = 967,242) and analyse grade point averages, secondary education and tertiary education using linear regression and linear probability models with standard errors clustered within families.The association between parental separation and educational achievement is negative and has remained similar across the birth cohorts. Differences according to parental union type and socioeconomic family background are rather small. The stability of the association over time suggests that the consequences of parental separation on children’s education have not changed over time, and they do not depend much on parental union type or family background.

## Introduction

Much of the existing research reports a generally negative association between parental separation and children’s education (e.g. Bernardi & Radl, 2014; Gähler & Palmtag, 2015; Grätz, 2015). Increasingly, research has also started to document whether and how this association has changed over time or across cohorts (Dronkers & Härkönen, 2008; Kalmijn, 2024; Kreidl et al., 2017; Sigle-Rushton et al., 2005). In this rapidly expanding literature, however, there are essential unknowns concerning how the parental type of the union is associated with the consequences of separation. In particular, it is not known whether parental separation from a marital or cohabiting union is more consequential for the children. This study contributes to the literature by examining both marital and cohabiting unions and how the association between parental separation and child outcomes has changed over time.

We study the dynamics between parental separation and educational achievement in Finland, which presents an interesting context since the country is a forerunner in the changes in partnerships associated with the second demographic transition. Non-marital cohabitation has become an increasingly common union type, and separation and divorce are increasingly common life events, each of which has become broadly socially accepted. Therefore, it could be assumed that the association between family transitions, such as parental separation, and child outcomes, including children’s education, has weakened over time (Härkönen et al., 2017). However, previous studies do not seem to support this assumption: most studies find that the association has remained similar over time (Dronkers & Härkönen, 2008; Kalmijn, 2024; Sigle-Rushton et al., 2005), and one study even found strengthening associations (Kreidl et al., 2017). However, none of the studies have analysed the association with both cohabitation and marriage.

In this study, we ask how parental separation is related to children’s education and whether the association has changed across birth cohorts. We use rich Finnish total population register data and child cohorts from 1987 to 2003, 1999, or 1995, depending on the outcome. We add to the existing literature by first examining how parental separation from marriage or cohabitation is associated with children’s education. Second, we examine how the association between parental separation and children’s educational achievement has changed across child cohorts. Third, we examine whether the association differs based on different educational outcomes. Fourth, we examine whether socioeconomic family background (parental education) moderates the association.

## Background

Previous research has generally found that children who have experienced parental separation fare less well in various outcomes. For instance, they have, on average, more mental health problems (e.g. Amato & James, 2010; Gähler & Palmtag, 2015), lower levels of education (e.g. Bernardi & Radl, 2014; Gähler & Palmtag, 2015; Grätz, 2015) and a higher risk of union dissolution (e.g. Härkönen et al., 2017; Kailaheimo-Lönnqvist et al., 2021; Wolfinger, 2005). Scholars have proposed many economic and social reasons for these associations, such as stress related to family conflict and the weakening of family resources due to separation, when children often live in single-parent households and benefit less from the resources of non-resident parents.

Parental separation is rather common, and many children experience it during childhood. In Finland, approximately 35 per cent of children experience parental separation (either from cohabitation or marriage) before the age of 16. Parental separation is much more common when the parents live in a non-marital cohabitation arrangement. In our study of cohorts born in the years 1987–2003, half of children whose parents were cohabiting when the children were born had experienced parental separation by the age of 16, whereas the proportion was one-third for children whose parents were married at some point during their childhood (authors’ own calculations). For all marriages, about 40 per cent of the marriages end in divorce in Finland and in the USA, the similar number was nearly 50 per cent (Raley & Sweeney, 2020; Statistics Finland, 2019). Thus, the separation rate in Finland is lower for married couples.

This study examines how parental separation is related to children’s education and how the association has changed across child cohorts. When parents separate, the child will likely lose some social and economic resources. Perhaps the most apparent lost resource is the double-income household since, after the dissolution of the union, the child will often live in a single-income household, and previous research suggests that single-income households are at an increased risk of poverty (Hübgen, 2018; Smock et al., 1999). However, the importance of lost economic resources may nonetheless be relatively small in a country like Finland, where the state provides welfare support based on a universalistic approach (Jalovaara & Andersson, 2018; Esping-Andersen, 1999) and education is free of charge, which causes it to differ from other more liberal and less generous welfare states, such as the United States (more in Finnish context -section).

As mentioned earlier, much prior research has linked parental separation to various adverse child outcomes, such as mental health problems and lower levels of education (Amato, 2001; Amato & James, 2010; Bernardi & Radl, 2014; Gähler & Palmtag, 2015; Grätz, 2015; Kiernan & Mensah, 2009; Mandemakers & Kalmijn, 2014). Thus, our initial hypothesis is based on the following assumption:

*H1: Parental separation is negatively associated with children’s educational achievement (general hypothesis).*

To what extent are child cohorts affected by changes over time? Finland is a forerunner in the partnership dynamics associated with the second demographic transition (Guzzo, 2014; Lesthaeghe, 2010). As non-marital cohabitation, non-traditional family forms, and separations have become increasingly common, the social stigma previously attached to them has diminished (Lansford, 2009). Additionally, children’s living arrangements after parental separation have evolved, with joint physical custody becoming more popular (Hakovirta et al., 2023). Research suggests that children and parents in shared physical custody typically show better outcomes (e.g. Augustijn, 2023; Riser et al., 2023; Baude et al., 2016; Nielsen, 2018; Steinbach, 2019; see the “Finnish Context” section for more details). Therefore, it could be expected that the association between parental separation and children’s education has weakened over time in Finland, given the likely improvement in the general well-being of children and parents in separated families.

However, previous research from various Western countries has generally found that the association between parental separation and children’s education has remained consistent over time (Dronkers & Härkönen, 2008; Kalmijn, 2024; Sigle-Rushton et al., 2005). One study even reported a strengthening association (Kreidl et al., 2017). The authors suggest that this strengthening may be due to stronger selection for family stability, meaning that more ‘low conflict’ couples are separating, which could lead to the negative consequences of separation outweighing the positive ones (Kreidl et al., 2017). In the Finnish context, where long-term cohabitation and other non-traditional family forms are more common than in many other countries, and considering that most previous studies from Western countries have not found any significant change over time, we propose two competing hypotheses:

*H2a: The association between parental separation and a child’s educational achievement has weakened across child cohorts (weakening over time hypothesis).*

*H2b: The association between parental separation and a child’s educational achievement has remained similar across child cohorts (no changes over time hypothesis).*

Despite Finland being a leader in the second demographic transition, notable differences still exist between cohabitation and marriage. Most marriages in Finland begin with cohabitation (Jalovaara, 2012), but couples who continue to cohabit without marrying often occupy lower socioeconomic positions and experience higher levels of union instability compared to those who eventually marry (Jalovaara, 2013; Jalovaara & Kulu, 2018; Perelli-Harris & Lyons-Amos, 2016; Kalmijn, 2013; Härkönen & Dronkers, 2006; Wood, Neels, & Kil, 2014). Even in the Nordic countries, married couples report higher levels of commitment and partnership quality than cohabiters (e.g. Wiik et al., 2009), which may contribute to a lower risk of separation. However, the higher threshold for separating among married couples could also lead to greater family conflict experienced by a child before parental separation. In general, cohabiters report lower levels of well-being (Soons et al., 2009) and tend to hold less traditional and familistic attitudes and values (Surkyn & Lesthaeghe, 2004). Despite these differences, previous research has shown that the factors leading to union dissolution are similar for both cohabiting and married couples (Jalovaara, 2013). However, there are legal distinctions between separating from marriage and from cohabitation. To divorce, one must apply to the district court, which includes a six-month reconsideration period and requires an agreement on the distribution of property. In contrast, separating from cohabitation only requires the individuals to move apart. When a couple has children, they must, regardless of their union type, agree on guardianship, living arrangements, and visitation rights (More information in the “Finnish context” section).

An Italian study found that children of cohabiting parents have lower educational attainment than those of married parents (Guetto & Panichella, 2019). However, when analysing the exposure to marriage, the study suggested that social selectivity might drive the adverse effects of cohabitation. Specifically, children born to families where the parents were married a few years before having a child were the most educationally advantaged, followed by children whose parents married after having a child (Guetto & Panichella, 2019). This indicates a clear selection process regarding marriage and its timing, linked to children’s educational attainment. In summary, previous studies suggest that children of married parents have “more to lose” when considering parental separation because their expected educational attainment is generally higher than that of children of cohabiting parents (this will be discussed in more detail when addressing family background effects). Thus, in the Finnish context, the third hypothesis is based on the following assumption:

*H3: The association is stronger for children from married families than those from cohabiting families (union-type hypothesis).*

The consequences of parental separation may also depend on socioeconomic family background and available resources (e.g. Grätz, 2015; Prix & Erola, 2017). According to the theory of maximally and effectively maintained inequality (Lucas, 2001; Raftery & Hout, 1993), intergenerational educational inequality is persistent because individuals with advantaged family backgrounds can better access advantageous educational options than their peers from less advantaged family backgrounds. Consequently, the effect of parental separation on the child’s education may be especially pronounced for children from advantaged family backgrounds, as they have the most to lose in terms of (potential) resources. In contrast, the cumulative disadvantage theory (O’Rand, 2009) posits that parental separation is most adverse for children from less advantageous family backgrounds. When negative life events or a shortage of resources accumulate, this accumulation negatively affects children’s achievements. Previous research on parental separation has found support for both theories, with some studies finding the most adverse effects are experienced by children from advantaged family backgrounds (e.g. Bernardi & Boertien, 2016; Bernardi & Radl, 2014; Biblarz & Raftery, 1999; Bussemakers et al., 2022; Guetto et al., 2022) and others finding such negative effects among those from disadvantaged family backgrounds (e.g. Augustine, 2014; Grätz, 2015; Lindemann, 2024; Mandemakers & Kalmijn, 2014; Nilsen et al., 2020; Schulz, 2022).

Additionally, another potential explanation for the differing impacts of parental separation based on parental education is that the effect of separation may vary according to the selectivity of the educational outcome. For less selective outcomes, such as the completion of secondary education in Finland, children from less advantaged backgrounds are more sensitive to parental separation (Bernardi & Comolli, 2019; Guetto & Panichella, 2019). Conversely, for more selective outcomes, such as university education, parental separation may matter more for children of highly educated parents (e.g. Guetto & Panichella, 2019). Furthermore, in terms of the intergenerational transmission of education, the association between parental separation and a child’s education is weakest for outcomes that are either very rare or very common (Bernardi & Comolli, 2019). This suggests that it is unlikely for children with low-educated parents to achieve a university education, so the added impact of parental separation does not generally alter that trajectory. Similarly, for very common outcomes, such as completing secondary education—where about 80 percent of individuals in Finland achieve this by age 20—parental separation is less significant because most children complete it regardless. Based on these considerations, we propose two competing hypotheses regarding family background:

*H4a: Parental separation is more strongly (negatively) associated with a child’s education when the child has an advantaged family background compared to children from a less advantaged family background because parental separation interferes with social inheritance (advantaged family background hypothesis).*

*H4b: Parental separation is more strongly (negatively) associated with a child’s education when the child has a less advantaged family background compared to children from an advantaged family background because of the accumulation of disadvantages (less advantaged family background hypothesis).*

### Finnish Context

#### Educational System

Finland is a strongly egalitarian welfare state with extensive income transfers for those in need and tuition-free education up to tertiary level. Children typically attend comprehensive school (primary and lower secondary education) from ages 7 to 16, following a mostly uniform curriculum. After comprehensive school, students proceed to secondary education, which lasts approximately three years and offers both vocational and general upper-secondary tracks. Upon completing secondary education, students can continue to tertiary education, which includes universities and universities of applied sciences. During the study period, the only significant educational change was the establishment of universities of applied sciences in 1991. However, since the oldest child cohort in this study was born in 1987, the new tertiary education route is unlikely to have caused differences across cohorts.

#### Separation from Marriage and Cohabitation

There were no significant changes in family law during the study period, which covers children born between 1987 and 2003. The main change occurred in 1988 when no-fault divorce was introduced, making it easier to obtain a divorce (Statistics Finland, 2022b). Since then, there have been no significant changes in family law for different-sex couples.

To separate from marriage, one must apply for divorce through the district court, which usually includes a six-month reconsideration period. Individuals must also agree on the distribution of property. In contrast, a cohabitation is, in principle, ended by just moving apart. However, if the couple has children, regardless of their union type, parents must agree on guardianship, maintenance, housing, and visitation rights. If conflicts arise regarding these issues, they are handled similarly for both married and cohabiting couples.

**Fig. 1:**
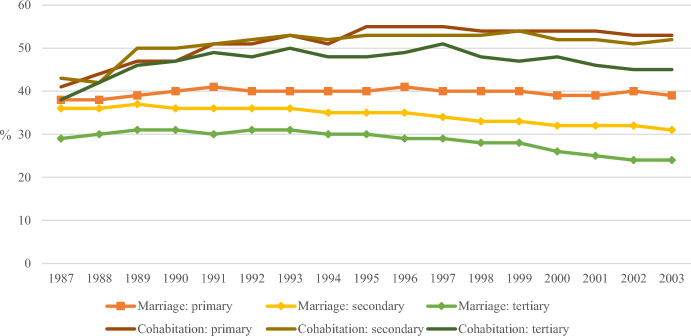
The proportion of children who experienced parental separation (age 0–15), whether from cohabitation or marriage, by child’s year of birth and parental education

The trends in the risk of separation differ between cohabiting and married couples (Fig. 1). Figure 1 shows that the proportion of children whose parents separated is lowest when the parents have a tertiary level of education and highest when parents have only a basic (or lower) level of education. This pattern holds true for both married and cohabiting couples. Notably, while the overall separation rate has not changed much over time (Fig. 8, Appendix), educational differences have widened. For the most recent cohorts, the risk of experiencing parental separation is more than double for children with lower-educated cohabiting parents compared to those with higher-educated married parents. Additionally, the likelihood of marriage has decreased (Statistics Finland, 2022a), suggesting that selection into marriage has likely become more pronounced. On the other hand, most children are now born outside of marriage: in 2020, 54 percent of all children were born to married parents, down from 75 percent in 1990 (Statistics Finland, 2021). Thus, there has been a clear increase in non-marital births during the study period.

In addition, the child’s mean age at parental separation has not changed much over time and children are about one year younger at parental separation from cohabitation compared to marriage, and children with highly educated parents older than children with less educated parents (Figs. 9 and 10, Appendix).

#### Children’s Living Arrangements After Parental Separation

Although there have been no changes in family law, there has been a significant shift in children’s living conditions after parental separation: joint physical custody has increased over time (Hakovirta et al., 2023). Joint physical custody means that many children now spend equal or nearly equal amounts of time with both parents, whereas in earlier years, parental separation typically resulted in children living primarily with their mother and having less frequent contact with their father. Joint physical custody (JPC) is associated with positive outcomes for both children and parents (e.g. Baude et al., 2016), although establishing causality is challenging. For instance, JPC has been linked to better co-parenting, fewer conflicts (Augustijn, 2023), improved psychological well-being, and reduced stress for children (e.g. Nielsen, 2018; Steinbach, 2019 review; Turunen, 2017). Additionally, some evidence suggests that joint physical custody is associated with higher levels of maternal well-being compared to sole physical custody (e.g. Augustijn, 2023; Riser et al., 2023). However, the causal relationship remains unclear, as many studies indicate that most children in joint physical custody arrangements have highly educated parents with low family conflict (e.g. Ortega-Gaspar et al., 2022). Nonetheless, joint physical custody allows children to benefit from the resources of both parents, strengthens the role of the father, and may enhance the well-being of both children and parents.

## Data and Methods

We use Finnish total population register data and focus on the grade point average (GPA) of child cohorts from 1987 to 2003, secondary education of child cohorts from 1987 to 1999 and tertiary education of child cohorts from 1987 to 1995. We restricted the sample to children whose parents lived together when children were born (and did not die during the childhood), and children born in Finland because the data comprise a full educational history only for those who attained educational degrees in Finland. This criterion results in a total of 967,242 children in the GPA sample, 782,657 children in the secondary education sample and 558,848 children in the tertiary education sample.

The child outcomes are (1) a grade point average (GPA), which is measured at the end of comprehensive school, i.e. lower secondary education, around ages 15 and 16 of age, (2) completion of secondary education by the age of 20 and (3) completion of or enrolment in tertiary education by the age of 26.

Parental separation from cohabitation and marriage arrangements was measured when the child was 0–15 years old (binary). Control and moderating variables include family income, sex, having a stepparent and highest parental education. Family income is the average total household income for a child between 1 and 15 years of age. Family income consists of all earnings and income transfers subject to state taxation in the household, such as universal child allowance, before taxes. It also includes any possible income of the stepparent for those years when the stepparent lived in the same household as the child. In the analysis, income is divided into family income deciles. As a measure of socioeconomic family background, we have used the highest parental education, which is categorical and measured when the child is between 1 and 15 years of age (basic education (refers to comprehensive education or less), secondary and tertiary education). Sex is a binary variable and living with a stepparent (at some point) is measured when the child is between 1 and 15 years of age (binary).

We analysed parental separations from cohabitation and marriage arrangements separately. Our married sample included children whose parents married before or during their childhood (although they may have cohabited before marriage). The cohabitation sample includes children whose parents cohabited and did not marry by the time the child was 15 years of age.

We used linear regression for GPA and linear probability models with robust standard errors (clustered on the family) to examine cohort differences in parental separation for binary outcomes such as secondary education and tertiary education. Linear probability models for binary outcomes were used because their estimates can be directly interpreted as probabilities (e.g. Gomila, 2021).

## Results

### Parental Separation and Children’s Education

As expected, parental separation was more common among children whose parents cohabited compared to those whose parents were married (Table 1). The parents’ educational level and family income were also higher in families where parents married. Having a stepparent was more common in families where the parents cohabited, which is partially explained by the higher union dissolution rate among cohabiting couples. Lastly, GPA as well as secondary education and tertiary education completion (or enrolment) rates are lower for children of cohabiting parents. The findings indicate that children whose parents are married generally are more advantaged.

**Table 1:** Descriptive statistics

		GPA		Secondary education		Tertiary education	
		Cohabitation (%)	Marriage (%)	Cohabitation (%)	Marriage (%)	Cohabitation (%)	Marriage (%)
Secondary education				73	82		
Tertiary education						40	54
Parental separation		51	33	51	34	49	35
Woman		50	49	49	49	50	49
Highest parental education							
	Basic	27	15	28	17	30	18
	Secondary	53	48	54	49	54	49
	Tertiary	20	37	18	34	16	32
Stepparent		25	21	26	21	27	22
Family income decile (mean, SD)		4.48 (2.12)	5.55 (2.28)	4.49 (2.12)	5.54(2.24)	4.42 (2.12)	5.53 (2.25)
GPA (standardised, mean/SD)		− 0.23 (1)	0.03 (1)				
*N*		122,338	844,904	93,161	689,496	60,372	498,476

Table 2 shows the association between parental separation and a child’s GPA. In Model 1, we find that the association is negative and is similar for children of cohabiting and married parents—just slightly stronger for children of married parents. The magnitude of the association, one fifth to one quarter of the standard deviation, is large. For example, it is larger than the difference in GPA for children with parents with a basic (or less) versus secondary education.

**Table 2:** The association between parental separation and a child’s GPA in lower secondary education; linear regression model with standard errors clustered within families

	Cohabitation		Marriage	
	M1	M2	M1	M2
Parental separation	− 0.215^***^	− 0.122^***^	− 0.256^***^	− 0.151^***^
	(0.006)	(0.006)	(0.002)	(0.003)
Woman	0.552^***^	0.552^***^	0.535^***^	0.536^***^
	(0.005)	(0.005)	(0.002)	(0.002)
Year of birth	0.013^***^	0.013^***^	0.009^***^	0.011^***^
	(0.001)	(0.001)	(0.000)	(0.000)
Parental education (ref.: basic)				
secondary	0.194^***^	0.141^***^	0.217^***^	0.165^***^
	(0.007)	(0.007)	(0.003)	(0.003)
tertiary	0.758^***^	0.585^***^	0.792^***^	0.605^***^
	(0.009)	(0.009)	(0.003)	(0.004)
Family income		0.085^***^		0.079^***^
		(0.001)		(0.001)
Stepparent		− 0.145^***^		− 0.146^***^
		(0.007)		(0.003)
Constant	− 28.027^***^	− 27.169^***^	− 18.907^***^	− 23.319^***^
	(1.191)	(1.166)	(0.439)	(0.430)
*N*	122,338	122,338	844,904	844,904

Standard errors in parentheses. * *p* < 0,05, ** *p* < 0,01, *** *p* < 0,001

Model 2 adds family income and the presence of a stepparent as controls. Both are influenced by separation itself and are best interpreted as potential mechanisms through which separation influences a child’s outcomes. A low family income and the presence of a stepparent are both associated with lower GPA. When controlling for these factors, the remaining association between parental separation and GPA is roughly halved compared to Model 1.

We also examined how parental separation is related to the education level achieved by children. We find that the association between parental separation and a child pursuing a secondary and tertiary education is negative, and it is slightly larger for children of married rather than cohabiting parents (Tables 3 and 4, Model 1). Similar to the GPA analysis, fully adjusted models show that low family income and the presence of a stepparent are associated with a lower likelihood of pursuing secondary and tertiary education and controlling for these variables weakens the association between separation and educational outcomes by approximately half (Tables 3 and 4, Model 2).

**Table 3:** The association between parental separation and a child’s secondary education; linear probability model with standard errors clustered within families.

	Cohabitation		Marriage	
	M1	M2	M1	M2
Parental separation	− 0.084^***^	− 0.047^***^	− 0.107^***^	− 0.069^***^
	(0.003)	(0.003)	(0.001)	(0.001)
Woman	0.050^***^	0.051^***^	0.034^***^	0.034^***^
	(0.003)	(0.003)	(0.001)	(0.001)
Year of birth	− 0.001^***^	− 0.001^***^	− 0.002^***^	− 0.002^***^
	(< 0.001)	(< 0.001)	(< 0.001)	(< 0.001)
Parental education (ref.: basic)				
secondary	0.109^**^	0.086^***^	0.099^***^	0.082^***^
	(0.004)	(0.003)	(0.002)	(0.003)
tertiary	0.206^***^	0.134^***^	0.174^***^	0.115^***^
	(0.004)	(0.004)	(0.002)	(0.002)
Family income		0.035^***^		0.025^***^
		(0.001)		(< 0.001)
Stepparent		− 0.056^***^		− 0.060^***^
		(0.007)		(0.001)
Constant	0.503	0.866	5.268^***^	3.878^***^
	(0.678)	(0.667)	(0.218)	(0.216)
*N*	93,161	93,161	689,496	689,496

Standard errors in parentheses. * *p* < 0,05, ** *p* < 0,01, *** *p* < 0,001

**Table 4:** The association between parental separation and a child’s tertiary education; linear probability model with standard errors clustered within families.

	Cohabitation		Marriage	
	M1	M2	M1	M2
Parental separation	− 0.083^***^	− 0.046^***^	− 0.106^***^	− 0.057^***^
	(0.004)	(0.004)	(0.002)	(0.002)
Woman	0.112^***^	0.113^***^	0.111^***^	0.112^***^
	(0.004)	(0.004)	(0.001)	(0.001)
Year of birth	− 0.005^***^	− 0.006^***^	− 0.009^***^	− 0.008^***^
	(0.001)	(0.001)	(< 0.001)	(< 0.001)
Parental education (ref.: basic)				
secondary	0.084^**^	0.062^***^	0.108^***^	0.084^***^
	(0.002)	(0.004)	(0.002)	(0.002)
tertiary	0.364^***^	0.290^***^	0.376^***^	0.286^***^
	(0.006)	(0.006)	(0.002)	(0.002)
Family income		0.039^***^		0.039^***^
		(0.001)		(< 0.001)
Stepparent		− 0.053^***^		− 0.062^***^
		(0.005)		(0.002)
Constant	9.760^***^	11.165^***^	18.575^***^	16.758^***^
	(1.528)	(1.507)	(0.514)	(0.506)
*N*	60,372	60,372	498,476	498,476

Standard errors in parentheses. * *p* < 0,05, ** *p* < 0,01, *** *p* < 0,001

### Trends Over Time

**Fig. 2:**
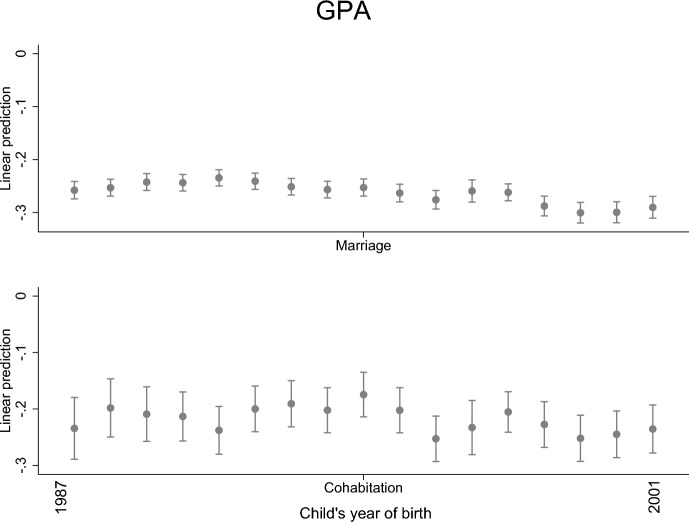
Parental separation and child’s GPA by child’s year of birth; linear regression model with standard errors clustered within families (models are adjusted for the highest parental education and sex)

**Fig. 3:**
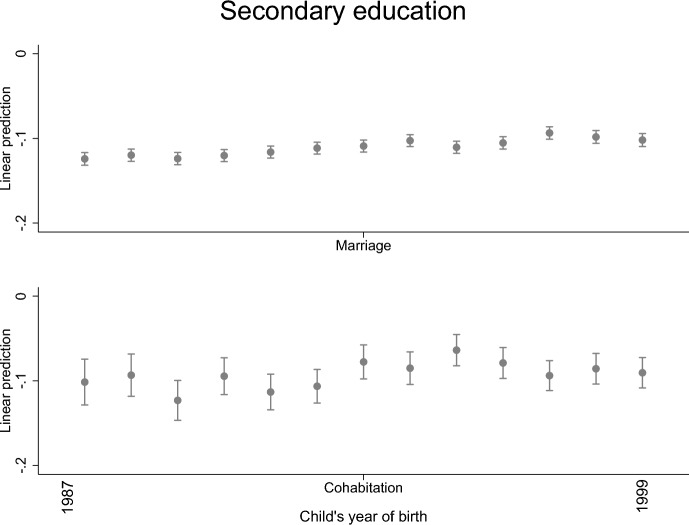
Parental separation and child’s secondary education level by child’s year of birth; linear probability model with standard errors clustered within families (models are adjusted for the highest parental education and sex)

**Fig. 4:**
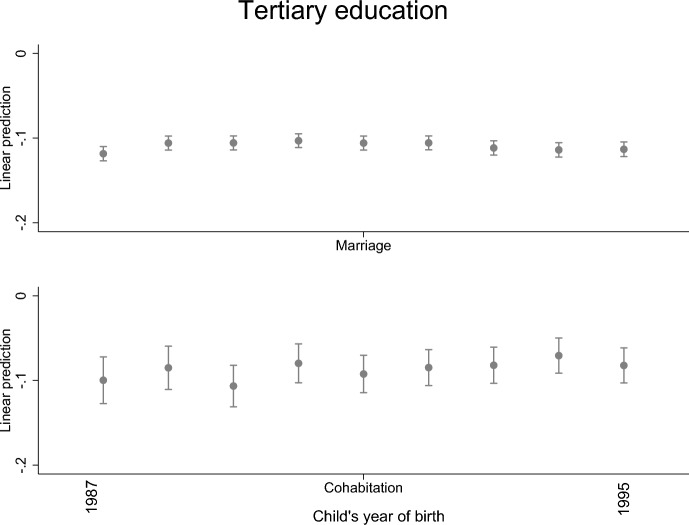
Parental separation and child’s tertiary education level by child’s year of birth; linear probability model with standard errors clustered within families (models are adjusted for the highest parental education and sex)

When examining changes across child cohorts, we find that the association between parental separation and a child’s GPA has remained notably similar both for children with married and cohabiting parents (Fig. 2). The findings were likewise similar regarding secondary and tertiary education (Figs. 3 and 4). The results are similar but slightly weaker when family income and stepparent are added to the models. Thus, inclusion or exclusion of them changes the interpretation (results not shown).

### Stratified Models by Socioeconomic Family Background

**Fig. 5:**
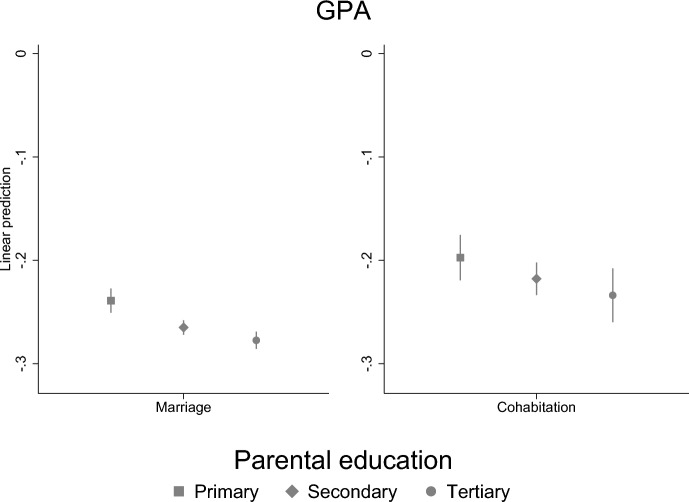
Parental separation and child’s GPA by parental education. Linear regression model with standard errors clustered within families (models are adjusted for sex)

**Fig. 6:**
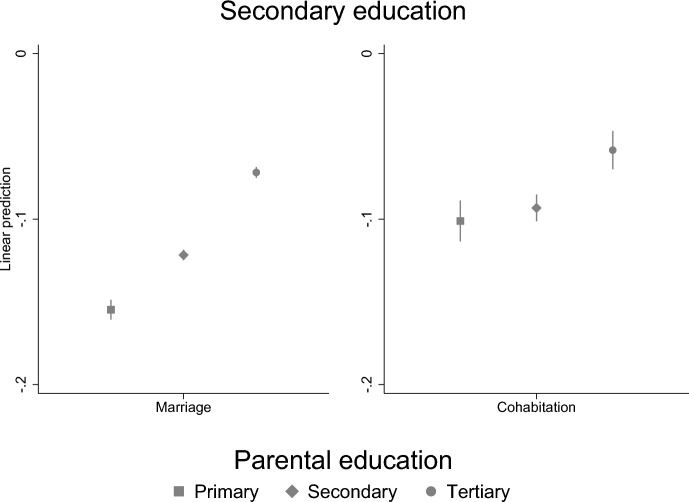
Parental separation and child’s secondary education by parental education. Linear probability model with standard errors clustered within families (models are adjusted for sex)

**Fig. 7:**
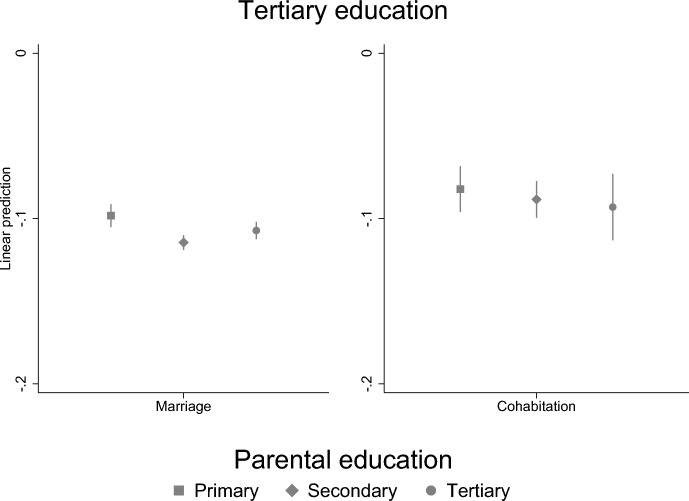
Parental separation and child’s tertiary education by parental education. Linear probability model with standard errors clustered within families (models are adjusted for sex)

**Fig. 8:**
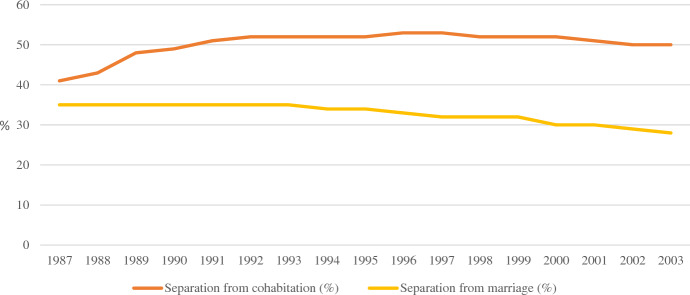
The proportion of children who experienced parental separation when parental separation occurred when the child was between 0 and 15 years of age (*N* = 967,418)

**Fig. 9:**
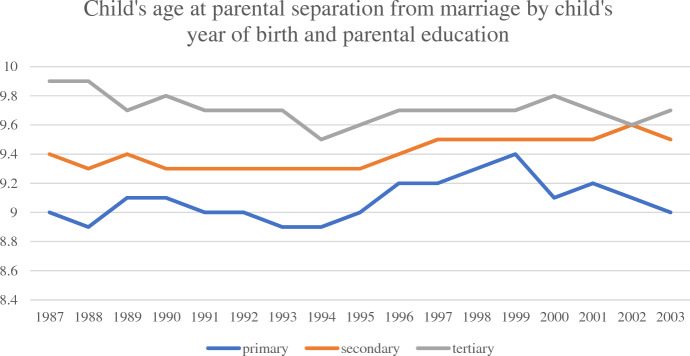
The mean age of children who experienced parental separation when parental separation from marriage occurred when the child was between 0 and 15 years of age

**Fig. 10:**
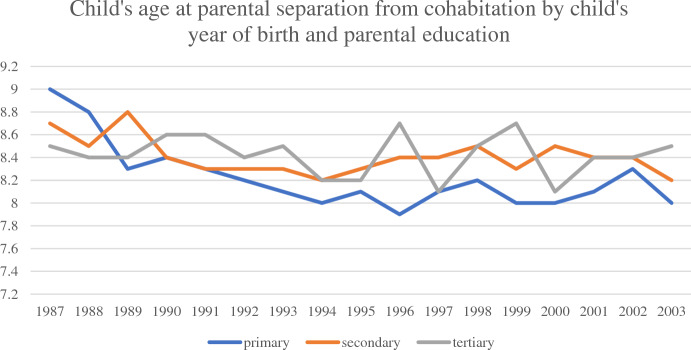
The mean age of children who experienced parental separation when parental separation from cohabitation occurred when the child was between 0 and 15 years of age

To examine how the association between parental separation and a child’s education may be different for different children, we conducted stratified analyses by socioeconomic family background (highest parental education). We find that parental separation is more strongly related to the GPA of highly educated parents’ children than children of less educated parents (Fig. 5). However, for children’s secondary education, the situation is the opposite: children of highly educated parents are less affected by parental separation than children of less educated parents (Fig. 6). In the tertiary level education, there are no differences by parental education (Fig. 7). Thus, our results suggest that even though children of highly educated parents have lower GPA, it does not interfere the completion of the secondary education which suggests that high parental education may compensate weaker educational success.

**Fig. 11:**
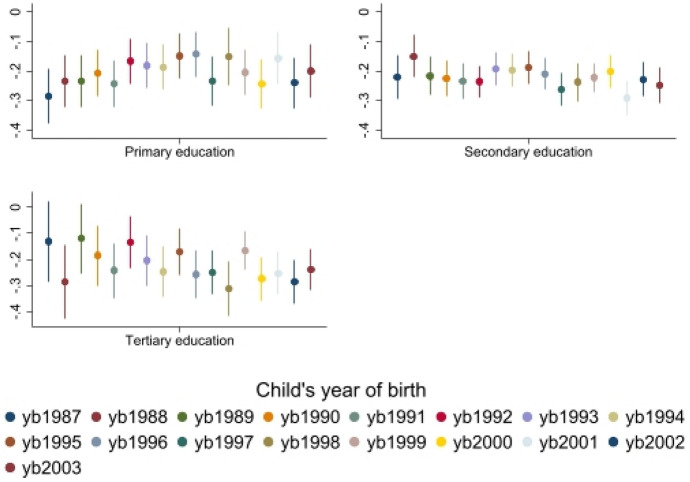
Parental separation from cohabitation and child’s GPA by child’s year of birth and parental education; linear regression model with standard errors clustered within families (models are adjusted for sex)

**Fig. 12:**
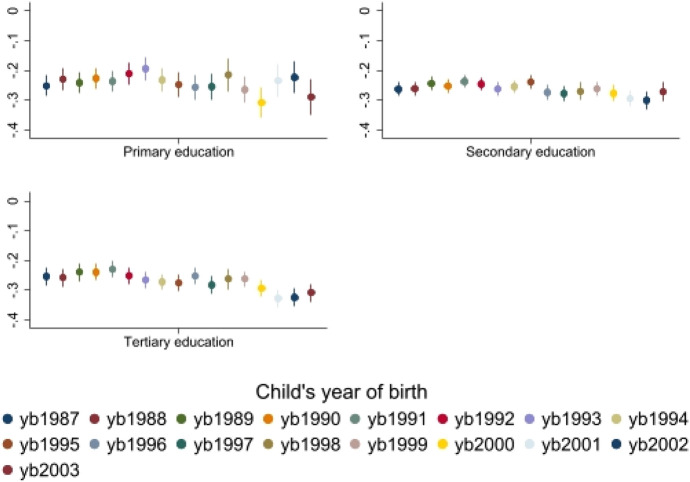
Parental separation from marriage and child’s GPA by child’s year of birth and parental education; linear regression model with standard errors clustered within families (models are adjusted for sex)

**Fig. 13:**
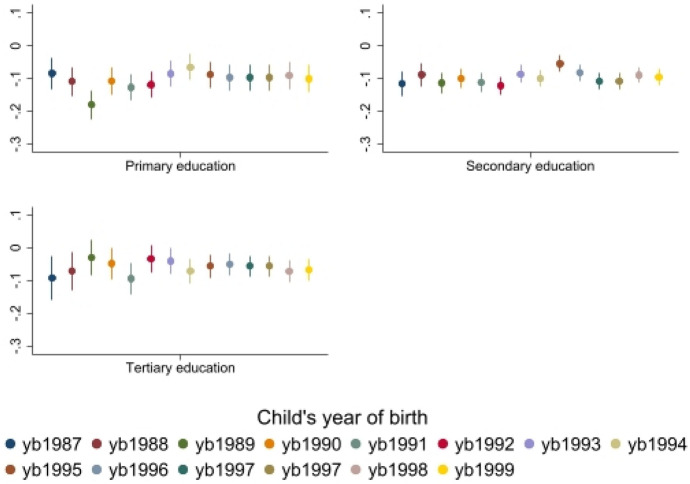
Parental separation from cohabitation and child’s secondary education by child’s year of birth; linear probability model with standard errors clustered within families (models are adjusted for sex)

**Fig. 14:**
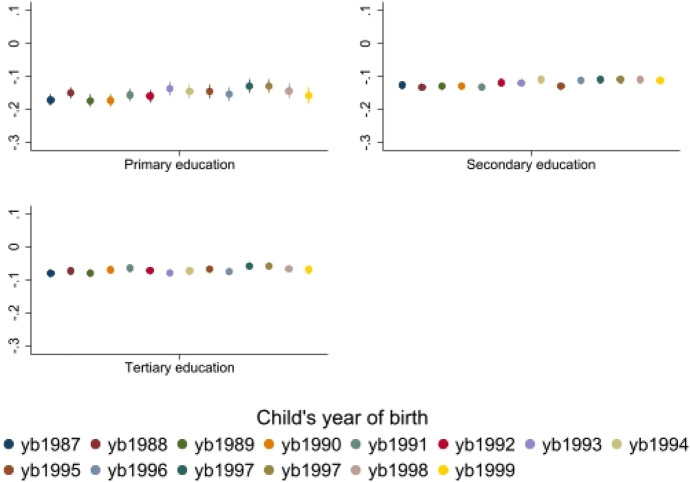
Parental separation from marriage and child’s secondary education by child’s year of birth; linear probability model with standard errors clustered within families (models are adjusted for sex)

**Fig. 15:**
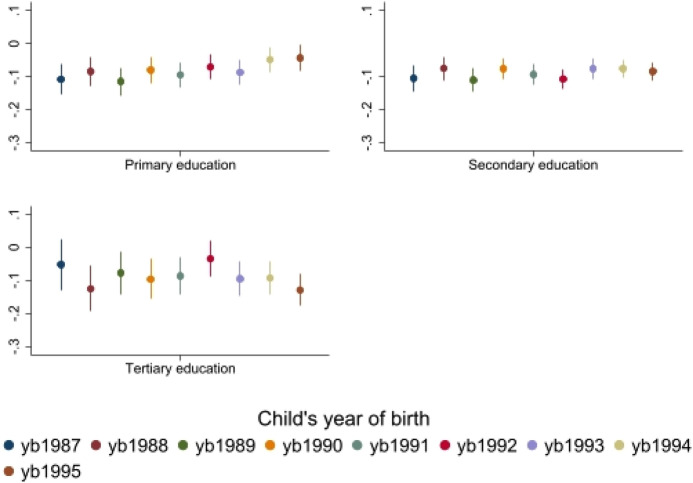
Parental separation from cohabitation and child’s tertiary education by child’s year of birth and parental education; linear probability model with standard errors clustered within families (models are adjusted for sex)

**Fig. 16:**
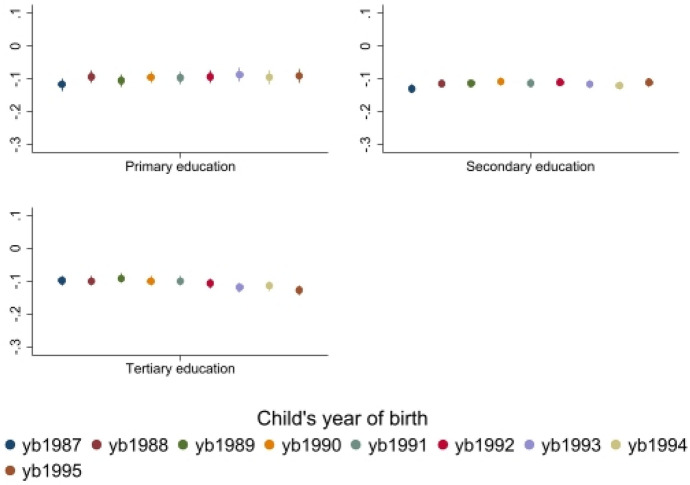
Parental separation from marriage and child’s tertiary education by child’s year of birth and parental education; linear probability model with standard errors clustered within families (models are adjusted for sex)

Our additional analysis also shows that that the association between parental separation and children’s education has remained quite similar across the child cohorts and the pattern is similar for both married and cohabiting parents (Figs. 11, 12, 13, 14, 15 and 16, Appendix).

### Support for Hypotheses

Thus, we find support for *Hypothesis 1*, which states that parental separation is negatively related to children’s educational achievement. Moreover, we do not find support for *Hypothesis 2a*, which states that the association has decreased over the years. Instead, we find support for *Hypothesis 2b*, which states that the association has remained similar over the years. Our results show that the association between parental separation and a child’s education has remained similar across child cohorts at all educational levels. We find partial support for *Hypothesis 3*, which states that the association is stronger for children with married parents compared to those with cohabiting parents—while a negative association is most clearly observed in the latter case, the differences between cohabiting and married parental arrangements are nonetheless negligible. Lastly, we find partial support for both *Hypotheses 4a* and *4b*. Parental separation is more strongly related to the GPA of highly educated parents’ children than children of less educated parents but for the completion of secondary education, it is the opposite: children of highly educated parents are less affected by parental separation than children of less educated parents. In the tertiary-level education, there are no differences by family background.

### Robustness Analysis

We examined whether the association depends on the age at which the child experiences the separation (Table 5, Appendix). The results are quite similar. They reveal that if parental separation occurs at an older age, the association weakens slightly, but overall, the timing of parental separation did not prove decisive. This indicates that the family stress, conflict and all issues related to parental separation drive the negative association between parental separation and child’s education, not only the timing of the separation.

We also conducted the main models using a different family structure variable. In the main models, we used separate dummy variables for parental separation and the presence of a stepparent. However, in the robustness analysis, we created a combined variable that includes both factors. The family structure variable, therefore, has the categories: ‘intact,’ ‘parental separation,’ and ‘parental separation + stepparent.’ The models clearly show that children who experience both parental separation and the introduction of a stepparent are more educationally disadvantaged compared to children who experience only parental separation, when compared to those from intact families (Appendix, Tables 6–8). This suggests that multiple changes in family structure are negatively associated with children’s educational outcomes.

**Fig. 17:**
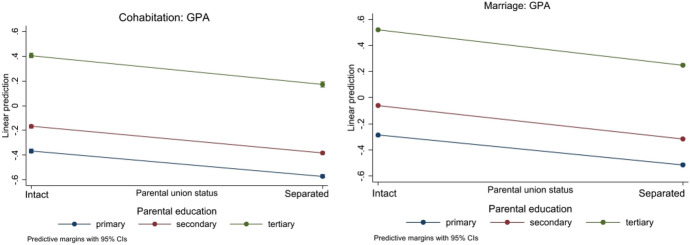
The association between parental separation and a child’s GPA by highest parental education

**Fig. 18:**
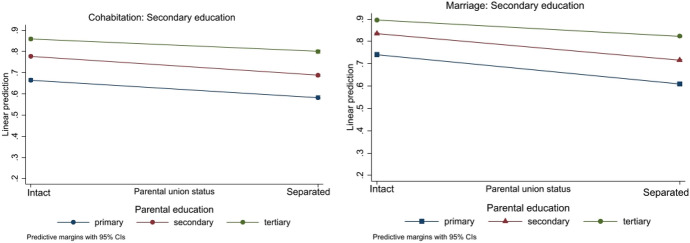
The association between parental separation and a child’s secondary education by highest parental education

**Fig. 19:**
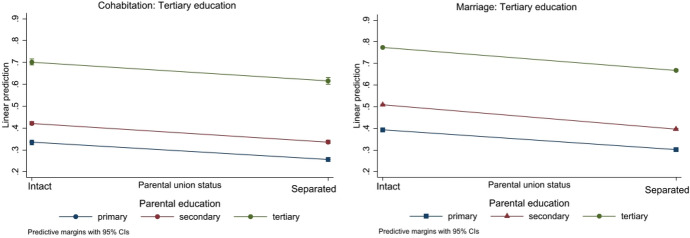
The association between parental separation and a child’s tertiary education by highest parental education

We examined linear predictions to ascertain whether parental separation is related to children’s education by relative and absolute outcomes. The results show that even though children who have experienced parental separation have a lower education than those who live in an intact family, parental separation only has a limited role in the intergenerational transmission of educational achievement, i.e. children with highly educated parents are also likely to be highly educated despite the parental separation, and vice versa (Figs. 17, 18, and 19 Appendix).

**Fig. 20:**
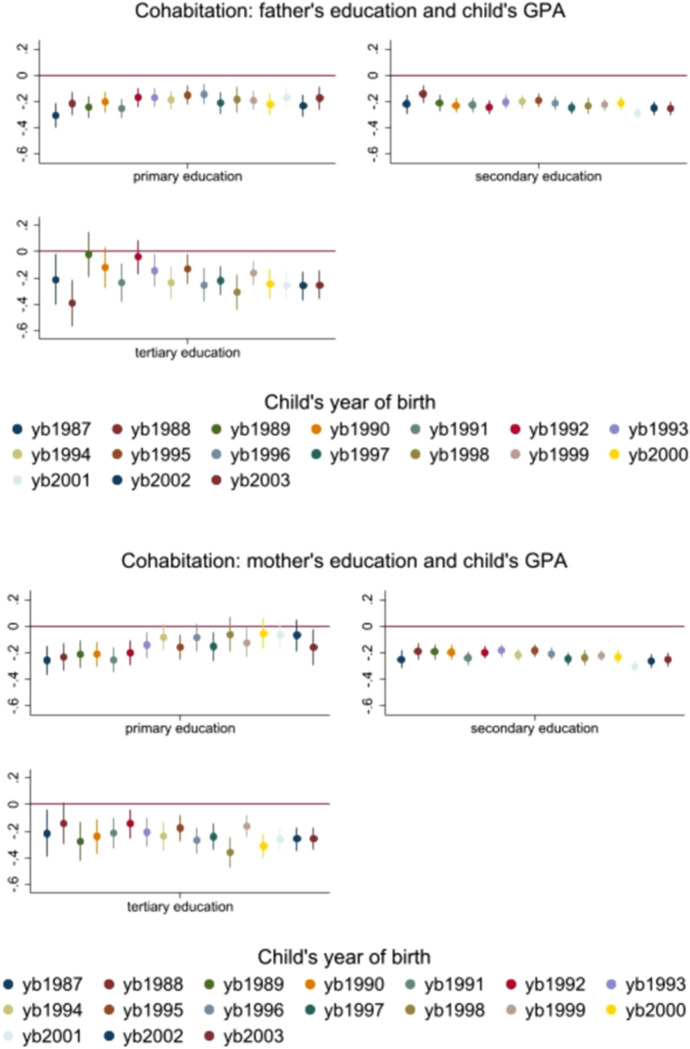

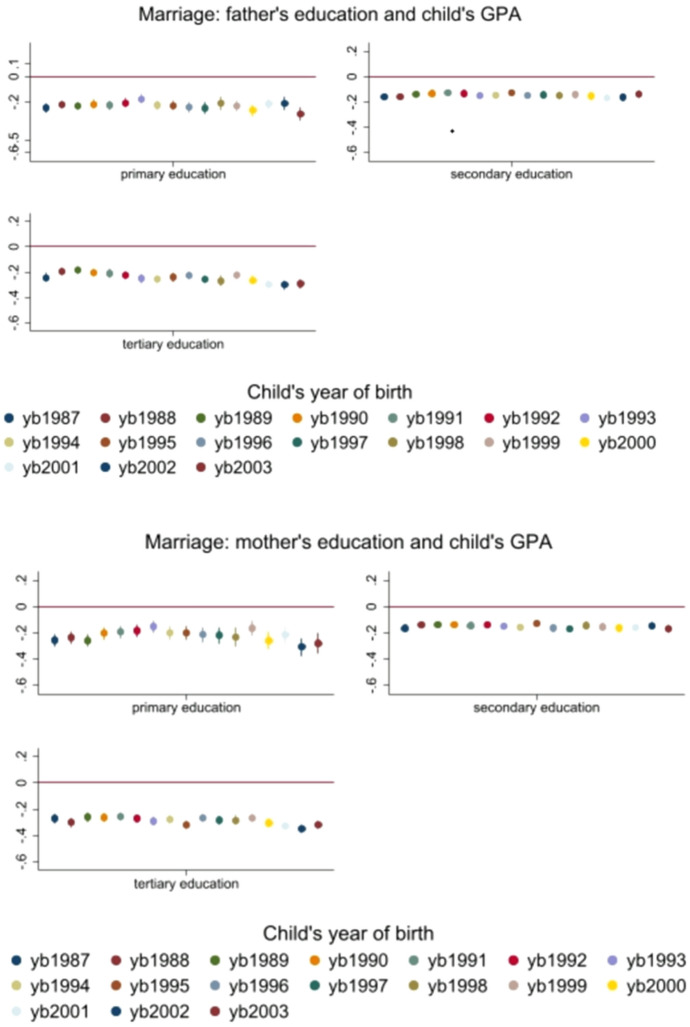
Parental separation and child’s GPA by parental education

**Fig. 21:**
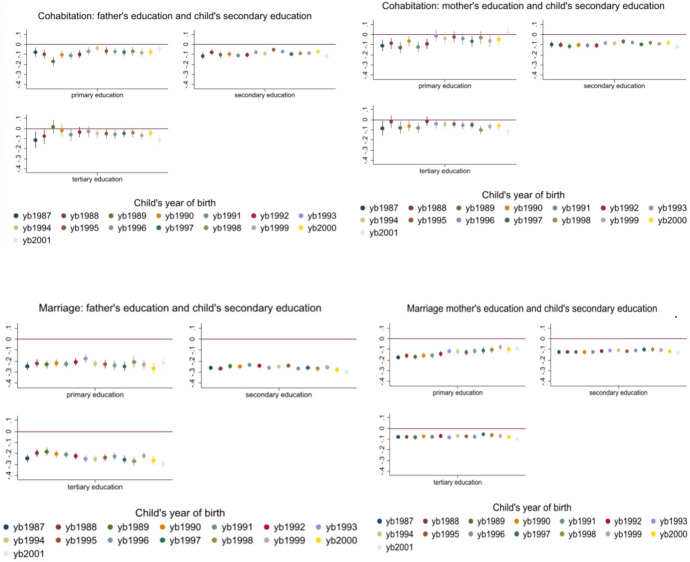
Parental separation and child’s secondary education by parental education

**Fig. 22:**
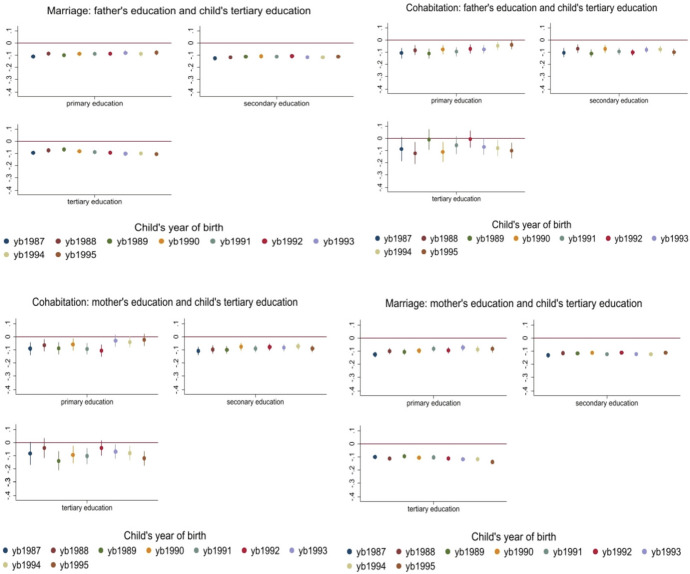
Parental separation and child’s tertiary education by parental education

Lastly, some previous research suggest that the measurements of family background may lead to differential findings (Bernardi & Boertien, 2017; Nilsen et al., 2020). Thus, we examined whether the results differ if we consider both the mother’s and father’s education separately, not just the highest parental education. Such a change in focus for the most part does not affect the results (Figs. 20, 21 and 22, Appendix), except with respect to children’s secondary education when parents separate from marriage: they show no differences at different educational levels, but paternal education is more strongly related to children’s secondary education than is maternal education.

## Discussion

This study assessed whether the association between parental separation and children’s education has changed over time across nearly two decades of child cohorts and whether the association is different in married and cohabiting parental unions. Previous research has found that children whose parents had separated fare less well on a variety of outcomes; for instance, they have more mental health problems, lower levels of education and a higher risk of union dissolution in adulthood (e.g. Gähler & Palmtag, 2015; Grätz, 2015; Kailaheimo-Lönnqvist et al., 2021). Moreover, only a few studies have examined how the association between parental separation and children’s education has changed over time (across cohorts). Most of the studies find that the association has remained similar (Dronkers & Härkönen, 2008; Kalmijn, 2024; Sigle-Rushton et al., 2005), and one study even found strengthening associations (Kreidl et al., 2017). The main contribution of this study is to examine these cohort changes both in separation from marriage and cohabitation.

We found, first, that parental separation is negatively associated with children’s education at all educational levels (GPA from lower secondary education, secondary education and tertiary education). Moreover, the association is similar at all educational levels; parental separation seems to decrease children’s secondary and tertiary education by 8.3 to 10.7 percentage points, and when controlling for a stepparent and family income it decreases by 4.6 to 6.9 percentage points. This can be interpreted to mean that some of the negative association between parental separation and children’s education is mediated by economic factors and changes in family structure.

Second, we found that the association is slightly stronger for children of married parents compared to children of cohabiting parents. This might be due to selection: couples that continue to cohabit often are in lower socioeconomic positions than those that eventually marry (Jalovaara, 2013; Jalovaara & Kulu, 2018), and thus parental separation may be more consequential for children with married parents because separation interferes with the intergenerational transmission of education. Our results also support this interpretation since we found some differences by socioeconomic family background especially among children of married parents. Our results similar with Italian study which found that children from cohabiting unions have weaker educational success than children from married unions (Guetto & Panichella, 2019).

Third, we found that the association between parental separation and children’s education has remained similar across cohorts. Thus, our study is in line with the majority of the previous studies, which have found that the association has remained similar over time (Kalmijn, 2024; Dronkers & Härkönen, 2008; Sigle-Rushton, Hobcraft, & Kiernan, 2005). Härkönen et al. (2017) explain the stability of the association by highlighting that even though some factors associated with parental separation, such as social stigma, previously attached to separation may have faded, other consequences, such as family conflict and loss of economic and social resources, may have remained stable (e.g. Pryor & Rodgers, 2001). Another potential explanation refers to changing the selection criterion to that of separation because, for example, parental separation has become increasingly associated with low levels of education (Härkönen & Dronkers, 2006; Härkönen et al., 2023). Thus, according to Härkönen and Dronkers (2006) the changing selectivity of parental separation can offset any weakening trend in its effects. The descriptive statistics partly support this interpretation: the separation rate itself has not changed over time, but the educational differences have grown over time, and for the most recent cohorts the risk of experiencing separation is more than double for children with lower educated cohabiting parents compared to those with higher educated married parents. However, when we stratified analysis by family background (parental education) we did not find any notable differences. Thus, it seems that changing composition does not “hide” the changes in association.

Lastly, we found that the association between parental separation and children’s education differs by socioeconomic background (parental education) in child’s GPA and secondary level education, but not in tertiary level education. Our findings are in line with previous studies that find that the impact of parental divorce may differ by selectivity of the educational outcome—for less selective outcome children of less advantaged parents were more affected (Bernardi & Comolli, 2019; Guetto & Panichella, 2019). Parental separation is more strongly related to those children GPA who have highly educated parents, but for the secondary education the case if opposite: children of highly educated parents are less affected by parental separation than children of less educated parents. Thus, our study suggests that even though children of highly educated parents have lower GPA, it is not seen in the completion of the secondary education which suggests that high parental education may compensate for weaker educational success. Moreover, the results show that children of highly educated parents have higher GPA despite the parental separation.

As with all studies, our study has both strengths and weaknesses. The strength of our study is the reliability of the total population register data, which cover a long time period (especially for GPA and secondary education) and enable us to study educational outcomes at all levels. Importantly, the data enable us to examine changes over time and across cohorts. Another strength is that we were able to include both cohabitation and marriage in our analysis, which is increasingly important due to the commonness of cohabitation. The main limitations include the fact that the register data did not make it possible to examine and account for the parent–child relationship or quality of parenting, which may have a role in children's educational attainment. Thus, our study did not investigate all possible mechanisms, and our results should be interpreted as associations, not as causal relations. The generalisability of our findings to other countries should be treated with caution because countries differ greatly in terms of the educational system and welfare systems. In the future, it would be important to examine also whether the role of stepparent and possible stepchildren has changed over time.

To summarise, our study suggests that the changes in society (e.g. support, joint physical custody more common) have not alleviated the consequences of parental separation. Our findings show that the consequences of parental separation on children’s education have not changed across almost two decades of child cohorts .

## Appendix

See Figs. 8, 9, 10, 11, 12, 13, 14, 15, 16, 17, 18, 19, 20, 21 and 22 and Tables 5, 6, 7 and 8

**Table 5:** Parental separation by child’s age, until 35, and child’s education; linear regression and probability models with standard errors clustered within families

		Cohabitation	Marriage
GPA			
	Parental separation 0–15	− 0.218*** (0.006)	− 0.280*** (0.003)
	Parental separation 16–35	− 0.213*** (0.008)	− 0.214*** (0.004)
Secondary education			
	Parental separation 0–15	− 0.088*** (0.003)	− 0.114*** (0.001)
	Parental separation 16–35	− 0.073*** (0.004)	− 0.093*** (0.002)
Tertiary education			
	Parental separation 0–15	− 0.080*** (0.005)	− 0.117*** (0.002)
	Parental separation 16–35	− 0.088*** (0.005)	− 0.091*** (0.002)

*Models are adjusted for highest parental education, year of birth and sex*

**Table 6:** Parental separation and child’s GPA. Linear regression models with standard errors clustered within families

	Marriage		Cohabitation	
	M1	M2	M1	M2
Family structure (ref. intact)				
parental separation	− 0.197^***^	− 0.141^***^	− 0.158^***^	− 0.108^***^
	(0.003)	(0.003)	(0.007)	(0.007)
parental separation + Stepparent	− 0.351^***^	− 0.280^***^	− 0.312^***^	− 0.255^***^
	(0.003)	(0.003)	(0.007)	(0.007)
Woman	0.540^***^	0.540^***^	0.552^***^	0.553^***^
	(0.002)	(0.002)	(0.005)	(0.005)
Year of birth	0.009^***^	0.011^***^	0.013^***^	0.013^***^
	(0.000)	(0.000)	(0.001)	(0.001)
Parental education (ref. basic)				
secondary	0.214^***^	0.178^***^	0.189^***^	0.146^***^
	(0.003)	(0.003)	(0.007)	(0.007)
tertiary	0.787^***^	0.621^***^	0.752^***^	0.595^***^
	(0.004)	(0.004)	(0.009)	(0.009)
Family income		0.079^***^		0.084^***^
		(0.001)		(0.001)
Constant	− 19.241^***^	− 23.411^***^	− 27.614^***^	− 27.529^***^
	(0.447)	(0.439)	(1.196)	(1.174)
*N*	844,904	844,904	122,338	122,338

Standard errors in parentheses. ^*^ *p* < 0.05, ^**^ *p* < 0.01, ^***^ *p* < 0.001

**Table 7:** Parental separation and child’s secondary education. Linear probability models with standard errors clustered within families

	Marriage		Cohabitation	
	M1	M2	M1	M2
Family structure (ref. intact)				
parental separation	− 0.086^***^	− 0.072^***^	− 0.068^***^	− 0.050^***^
	(0.001)	(0.001)	(0.003)	(0.003)
parental separation + Stepparent	− 0.147^***^	− 0.129^***^	− 0.124^***^	− 0.103^***^
	(0.002)	(0.002)	(0.004)	(0.004)
Woman	0.033^***^	0.033^***^	0.049^***^	0.049^***^
	(0.001)	(0.001)	(0.003)	(0.003)
Year of birth	− 0.002^***^	− 0.001^***^	0.000	0.000
	(0.000)	(0.000)	(0.000)	(0.000)
Parental education (ref. basic)				
secondary	0.082^***^	0.072^***^	0.097^***^	0.080^***^
	(0.002)	(0.002)	(0.004)	(0.004)
tertiary	0.154^***^	0.111^***^	0.191^***^	0.130^***^
	(0.002)	(0.002)	(0.004)	(0.004)
Family income		0.021^***^		0.033^***^
		(0.000)		(0.001)
Constant	3.911^***^	3.016^***^	1.142	1.101
	(0.218)	(0.216)	(0.679)	(0.669)
*N*	779,776	779,776	110,076	110,076

Standard errors in parentheses. ^*^ *p* < 0.05, ^**^ *p* < 0.01, ^***^ *p* < 0.001

**Table 8:** Parental separation and child’s tertiary education. Linear probability models with standard errors clustered within families

	Marriage		Cohabitation	
	M1	M2	M1	M2
Family structure (ref. intact)				
parental separation	− 0.080^***^	− 0.051^***^	− 0.062^***^	− 0.041^***^
	(0.002)	(0.002)	(0.005)	(0.005)
parental separation + Stepparent	− 0.151^***^	− 0.114^***^	− 0.118^***^	− 0.094^***^
	(0.002)	(0.002)	(0.005)	(0.005)
Woman	0.113^***^	0.113^***^	0.113^***^	0.115^***^
	(0.001)	(0.001)	(0.004)	(0.004)
Year of birth	− 0.009^***^	− 0.009^***^	− 0.005^***^	− 0.006^***^
	(0.000)	(0.000)	(0.001)	(0.001)
Parental education (ref. basic)				
secondary	0.105^***^	0.087^***^	0.081^***^	0.064^***^
	(0.002)	(0.002)	(0.005)	(0.004)
tertiary	0.374^***^	0.291^***^	0.361^***^	0.293^***^
	(0.002)	(0.002)	(0.006)	(0.006)
Family income		0.039^***^		0.039^***^
		(0.000)		(0.001)
Constant	18.329^***^	17.151^***^	10.239^***^	11.016^***^
	(0.522)	(0.515)	(1.548)	(1.527)
*N*	498,476	498,476	60,372	60,372

Standard errors in parentheses. ^*^ *p* < 0.05, ^**^ *p* < 0.01, ^***^ *p* < 0.001
